# *Rickettsia felis* Infection, Tunisia

**DOI:** 10.3201/eid1201.050876

**Published:** 2006-01

**Authors:** Abir Znazen, Jean-Marc Rolain, Nader Hammami, Adnane Hammami, Mounir Ben Jemaa, Didier Raoult

**Affiliations:** * Université de la Méditerranée, Marseille, France;; † Universitaire Habib Bourguiba, Sfax, Tunisia;; ‡ Universitaire Hédi Chaker, Sfax, Tunisia

**Keywords:** *Rickettsia felis*, serology, Tunisia, dispatch

## Abstract

We report, for the first time, serologic evidence of *Rickettsia felis* and *R*. *aeschlimannii* infections acquired in Tunisia from 1998 to 2003. We found that most patients with antibodies against both *R*. *conorii* and *R*. *typhi* had serologic evidence of *R*. *felis* infection.

Rickettsioses are arthropod-borne zoonoses with geographic distributions determined by the ecology of their vectors. The genus *Rickettsia* is divided into 2 groups, the spotted fever group (SFG) and the typhus group (TG), mainly based on their intracellular positions, optimal growth temperatures, gas chromatographic proportion (%), DNA content, clinical features, epidemiologic aspects, and antigenic characteristics. Recently, a new species of *Rickettsia* that infected humans, *R*. *felis*, was reported (*1*), and the whole genome of this species has recently been sequenced (*2*). The pathogenic role of *R*. *felis* in humans was demonstrated first in Texas (*1*), with subsequent reports of this "fleaborne spotted fever" confirmed in patients from Europe and South/Central America (*3**–**5*) by polymerase chain reaction (PCR), serologic tests, or both. In Tunisia, North Africa, the epidemiology of rickettsial diseases has not been documented and only 1 study concerning these diseases has been conducted. The study, conducted in 1995, confirmed that *R*. *conorii* and TG rickettsia were endemic in Tunisia, with estimated antibody prevalences of 9% and 3.6%, respectively (*6*). The aim of our study was to investigate the diversity of rickettsioses in Tunisia through the use of serologic assays.

## The Study

Serum samples obtained from patients suspected to have clinical rickettsial infection (fever associated with an eschar or cutaneous rash) were collected from 1998 to 2003 at the Laboratory of Microbiology CHU Habib Bourguiba, Sfax, Tunisia. Acute- and convalescent-phase serum samples, when available, were stored at –80°C until they were tested in a multiple-antigen immunofluorescence assay (IFA) at the Unité des Rickettsies Marseille (*7*). Ten SFG rickettsial antigens were used: *R*. *conorii conorii* strain 7, *R*. *africae* strain ESF-5, *R*. *sibirica mongolitimonae* strain HA-91T, *Rickettsia aeschlimannii* strain MC16T, *R*. *massiliae* strain Mtu1T, *R*. *helvetica* strain C9P9, *R*. *slovaca* strain 13-B, *R*. *conorii israelensis* strain ISTTCDC1, *R*. *felis* strain URRWXCal2 ATTC VR-1525, and *R*. *typhi* strain Wilmington. Antigens were produced at the Unité des Rickettsies Marseille as previously reported (*8*). Immunoglobulin G (IgG) antibody titers of 1:128, seroconversion in paired serum specimens, or IgM antibody titers of 1:32 against any species were considered evidence of recent *Rickettsia* infection (*7*).

Identification at the species level was determined by Western blot (WB) and cross-adsorption assays in accordance with procedures described elsewhere (*7**,**8*). *R. conorii*, *R*. *aeschlimannii*, *R*. *felis*, and *R*. *typhi* isolates were suspended briefly in sterile distilled water and adjusted to 2 mg protein/mL. Twenty microliters of the preparation was electrophoresed at 100 V for 2 h through a separating gel containing 12% polyacrylamide by means of a Mini-Protean II cell apparatus (Bio-Rad, Marnes la Coquette, France). A mixture of prestained molecular mass standards (Kaleidoscope; Bio-Rad) was used to estimate the molecular masses of the separated antigens. Resolved antigens were transferred onto a 0.45-μm pore nitrocellulose membrane (Bio-Rad) that was electrophoresed for 1 h at 4°C and 100 V. The blots were blocked overnight at 4°C with 5% nonfat milk powder in Tris-buffered saline (TBS) and were washed with distilled water. Serum specimens (diluted at a ratio of 1:200 in TBS with 3% nonfat milk powder) were applied to the blots for 1 h at room temperature. After three 10-min washes in TBS with 3% nonfat milk powder, the blots were incubated for 1 h with peroxidase-conjugated goat antihuman IgG (Southern Biotechnology Associates, Birmingham, AL, USA) diluted at a ratio of 1:750 in TBS with 3% nonfat milk powder. The blots were washed 3 times in TBS, and bound conjugate was shown by incubation in a solution of 0.015% 4-chloro-1-naphthol (Sigma, Lyon, France) and 0.015% hydrogen peroxide in TBS with 16.7% methanol for 15 min. WB analysis was done both before and after cross-adsorption in accordance with procedures described elsewhere (*8*). For patients with serologic evidence of *R*. *felis* infection, epidemiologic and clinical data were collected from medical records.

From 1998 to 2003, 753 serum samples were collected from 638 patients in Tunisia and sent to Marseille for analysis. Paired serum specimens were available for 115 patients. Serologic evidence of recent *Rickettsia* infection was found in 86 patients. Serum samples from 63 of these patients exhibited wide cross-reactive antibodies between SFG rickettsia and either *R*. *felis* (45 cases) or *R*. *typhi* (18 cases) (Figure); 19 serum samples had cross-reactive antibodies between *R*. *felis* and *R*. *typhi*. Finally, 3 samples had cross-reactive antibodies for SFG only (except *R*. *felis*), and 1 serum sample was positive for *R*. *typhi* only. WB with cross-adsorption analysis was performed for 21 serum samples, yielding species-level identification for 19 cases as follows: 3 *R*. *conorii*, 2 *R*. *aeschlimannii*, 6 *R*. *typhi*, and 8 *R*. *felis*.

**Figure:**
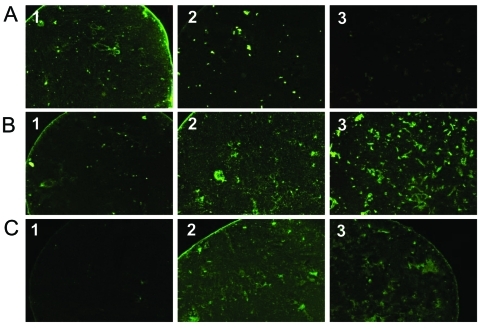
Pictures of immunofluorescence assay performed on serum specimens with proven *Rickettsia conorii* (A), *R*. *felis* (B), or *R*. *typhi* (C) infection showing cross-reactive antibodies. Antigens tested were *R*. *conorii* (column 1), *R*. *felis* (column 2), and *R*. *typhi* (column 3). The serum with *R*. *conorii* infection reacts with *R*. *conorii* and *R*. *felis* antigens but not with *R. typhi* (A). Conversely, the serum with *R. typhi* infection reacts with *R*. *typhi* and *R*. *felis* but not with *R*. *conorii* (C). Finally, the serum with *R*. *felis* infection reacts with *R*. *felis*, *R*. *conorii*, and *R*. *typhi*.

Clinical data were available for only 1 patient with *R*. *aeschlimannii* infection. This patient had fever with meningitis without inoculation eschar or cutaneous rash. Serologic and clinical characteristics were available for 8 patients with *R*. *felis* infection (Table); most of the patients lived in urban areas, and 1 came from Libya. All 8 patients had fever and a maculopapular rash, and none reported a history of flea or tick bite or had an eschar. Two patients had peripheral adenopathy on admission: cervical and inguinal in the first patient and axillary and inguinal in the second patient.

**Table:** Epidemiologic, clinical, and serologic data for patients with fleaborne spotted fever*

No.	Serologic titers (IgG/IgM)*									
	*Rickettsia conorii*	*R. felis*	*R. typhi*	Group	Locality	Animal contact	Fever	Eschar	Cutaneous rash	Other signs
2,120	1:128/1:64	1:128/1:16	0/0	2	Sfax	–	40°	No	MP	No
2,274	1:256/1:256	1:512/1:512	0/0	2	–	–	–	–	–	–
2,275	1:256/1:256	1:512/1:512	0/0	2	Moknine	Sheep/cows	40°	No	No	No
2,147	1:128/1:128	1:1,024/1:1,024	1:128/1,024	3	Sousse	Dogs/birds	40°	No	MP	Interstitial pneumopathy
2,245	1:256/1:64	1:1,024/1:512	1:128/1:512	3	Sfax	Cats/sheep	40°	No	MP	adenopathy
2,608	1:512/1:64	1:1,024/1:256	1:1,024/0	3	Sfax	No	Yes	No	MP	adenopathy
2,547	1:32/1:32	1:512/1:256	1:256/1:128	3	Libya	No	Yes	No	MP	
2,421	1:128/1:32	1:512/1:128	1:256/0	3	Sfax	No	Yes	No	MP	

*Ig, immunoglobulin; MP, maculopapular.

## Conclusions

In this study, IFA and WB identified *R*. *felis* infection in patients from Tunisia. Serologic cross-reactions are common among *Rickettsia* species in both the SFG and TG. A difference in specific IgG or IgM antibody titers has been useful for distinguishing murine typhus from epidemic typhus (*8*). More sophisticated serologic methods are needed to identify the causative agent at the species level (*9*). WB performed on 7 of 18 serum samples with cross-reactions between SFG, *R*. *felis*, and *R*. *typhi* confirmed the diagnosis of *R*. *felis* in 5 patients and TG rickettsia in 1 patient; the diagnosis remained undetermined in 1 patient. Recently, the whole genome of *R*. *felis* has been sequenced and demonstrated genetic similarity with *R*. *typhi* but showed some genes missing from the *R*. *conorii* genome (*2*). Thus, *R*. *felis* may be the major cause of cross-reactions between *R*. *typhi* and *R*. *conorii* or other tickborne spotted fever agents. Cross-reactions between the 2 groups of *Rickettsia* have been puzzling because this activity is not reported in experimentally infected guinea pigs and mice (*10*). In fact, we speculate that many of the reactions with both *R*. *typhi* and *R*. *conorii* are caused by *R*. *felis* infection. This hypothesis is supported by our findings of cross-reactivity in serum specimens from 5 of 7 patients with confirmed *R*. *felis* infection. Indeed, when antigens are not available, this cross-reactivity should be a good screening method for *R*. *felis* infection. Alternatively, all serum specimens exhibiting cross-reactivity between *R*. *typhi* and *R*. *felis* only were considered to be TG rickettsia infection after WB.

To the best of our knowledge, this is the first report of patients with *R*. *felis* and *R*. *aeschlimannii* infections in Tunisia. In Morocco, similar results have been reported (*11*). Several cases of SFG rickettsioses have been reported from North Africa, including 1 patient with *R*. *aeschlimannii* infection from Morocco (*12*) and 1 patient with *R*. *sibirica mongolitimonae* infection from Algeria (*13*). These results are not surprising since vectors of *R*. *felis* and *R*. *aeschlimannii* are present in North Africa (*14*). Indeed, *R*. *aeschlimannii* has been isolated from *Hyalomma marginatus* ticks collected from camels in Morocco (*15*), and *R*. *felis*–infected fleas in Algeria have been recently reported (*14*). Since *R*. *felis* has a worldwide distribution and infestation with these fleas is very common, *R*. *felis* and fleaborne spotted fever may occur worldwide.

Only a few human cases of *R*. *felis* infection diagnosed by serologic tests or PCR have been reported: 1 case from the United States (Texas) (*1*), 3 from Mexico (*3*), 2 from France, 2 from Brazil (*4*), and 2 from Germany (*5*). In the Texas case (*1*), the patient had clinical features similar to those associated with murine typhus. However, patients with *R*. *felis* and central nervous system and pulmonary involvement have been reported from Mexico (*3*). In our study, 1 of the patients with *R*. *felis* infection had pulmonary involvement and 2 had adenopathy. Although none had an eschar or a history of flea bite, 3 patients had contact with animals.

Our findings indicate the need for further studies to determine the distribution of *R*. *felis* and the prevalence of this agent and associated infection. These results suggest that fleaborne spotted fever, as well as other SFG rickettsioses, are common in Tunisia.
